# Influenza B virus suppression of Chromosome Y-linked genes increases pulmonary virus replication and disease severity in male mice

**DOI:** 10.21203/rs.3.rs-9066058/v1

**Published:** 2026-03-24

**Authors:** Sabal Chaulagain, Patrick Creisher, Han-Sol Park, Jennifer Liu, L. Claire Gay, Brittany Seibert, Aihui Wang, Miranda Jimenez, C. Joaquin Caceres, Daniel Perez, Sabra Klein

**Affiliations:** Johns Hopkins Bloomberg School of Public Health; Johns Hopkins Bloomberg School of Public Health; Johns Hopkins Bloomberg School of Public Health; University of Georgia; University of Georgia; Johns Hopkins Bloomberg School of Public Health; Johns Hopkins Bloomberg School of Public Health; Iowa State University; University of Georgia; Johns Hopkins Bloomberg School of Public Health

**Keywords:** plasmacytoid dendritic cells, oseltamivir, type I IFN, influenza, Chromosome Y, Chromosome X

## Abstract

Influenza B viruses (IBV) are transmitted among humans, with disease being worse in men than women. C57BL/6 male and female mice were inoculated with Victoria lineage B/Brisbane/60/2008 containing a PB2 F406Y mutation. Juvenile, adult, and aged males exhibited greater virus titers, morbidity, and pulmonary inflammation than age-matched females. Oseltamivir treatment reduced virus titers in males thereby reducing morbidity and pulmonary cytokine responses to female-equivalent levels. Infection of transgenic and mutant mice that allowed for separation of sex chromosome dosage from gonadal sex effects revealed that the presence of a Y chromosome (ChrY) was the major contributing factor for male-biased susceptibility to IBV. IBV infection suppressed pulmonary Uty and Ddx3y expression in ChrY-bearing mice, which was reversed by oseltamivir treatment, suggesting that virus replication inhibits protective ChrY gene expression, causing male-biased IBV pathogenesis.

## Introduction

The pathogenesis of many respiratory infectious diseases differs between sexes, reflecting differences in both the ability to control pathogen replication (i.e., resistance) and mitigate inflammation-induced pathology (i.e., tolerance) following infection.^1^ Biological sex differences in infectious disease outcomes can be caused by gonadal steroids, sex chromosome complement, and even anatomical differences between the sexes.^2^ Biological sex differences are distinct from but complementary to gender differences that are caused by societal or cultural norms, which in the context of infectious diseases might impact exposure to pathogens as well as utilization, acceptance, or access to healthcare and treatments.^3^ Combined, these factors can result in differences between the sexes in the pathogenesis of many infectious diseases, including those that have caused pandemics over the last century or more.^4, 5^

For seasonal influenza epidemics, annual cases are caused by influenza A viruses (IAVs; H1N1 and H3N2) and influenza B viruses (IBVs; Victoria and Yamagata).^6^ The transient, acute nature of influenza symptoms in most individuals, means that many cases of influenza-like illness are underdiagnosed and underreported, with sex and gender differences often not considered. Limited epidemiological data from several countries reveal that cases of severe disease that require hospitalization are greater in prepubertal and older aged males compared with age-matched females.^6, 7, 8, 9, 10, 11^ In China, for example, pneumonia and influenza death rates are elevated with older age, which is apparent among males aged 65–74 years but not among females until ages ≥75 years and older.^12^ Similar observations are reported in Hong Kong, in which among children and older adults, males experience more severe disease (i.e., are hospitalized) than age-matched females for both IAV and IBV.^13^ During the 2009 H1N1 pandemic, reproductive aged females were more likely to be hospitalized than age-matched males in diverse countries, including Canada, Australia, and Japan^14, 15, 16^, which continued to be reported in the years following the pandemic.^16^ Greater disease from 2009 H1N1 in adult females is replicated in mice and reflects greater tolerance rather than resistance to the virus that is mediated both by greater tissue repair^17^ and androgen receptor signaling^18^ in males than females. The human data illustrate that across diverse countries, social norms, and economies, sex and age differences in the severity of influenza are reported.^5^ Preexisting immunity and host-pathogen mechanisms of virulence also likely impact whether males or females are more or less susceptible to disease.

IBVs circulate only in humans and cause respiratory disease that has been understudied in part because these viruses lack pandemic potential, have a slower evolution rate, and their disease impact on the population is historically less than IAV. Prior to the COVID-19 pandemic^19^, there were two circulating lineages of IBV^20^ that caused more than 20% of confirmed influenza infections globally, with both lineages contained in the annual quadrivalent influenza vaccine.^21, 22^ After the COVID-19 pandemic, the Yamagata lineage appears to have gone extinct.^19^ Using mouse models, we sought to identify mechanisms of sex differences in the pathogenesis of IBV. We hypothesized that sex differences in IBV could be caused by differential resistance to infection, tolerance to immunopathology, or both mechanisms of virulence, which might involve the effects of either gonadal sex or sex chromosome complement.

## Results

### Adult males suffer worse outcomes from IBV infection

**Figure 1:**
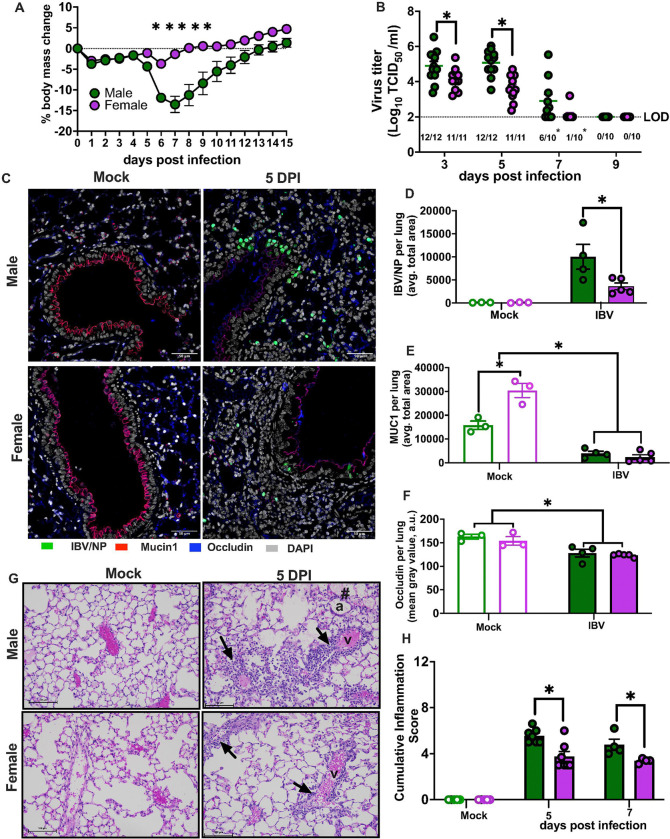
Adult males suffer worse outcomes from influenza B virus infection. Adult (8-weeks old) male (green) and female (purple) C57BL/6 mice were intranasally inoculated with 10^5^ TCID50 B/Brisbane/60/2008F406Y. For morbidity study, body mass changes were recorded daily up to 15 days post infection (dpi) (**A**). Subsets of mice were euthanized at 3, 5, 7, or 9 dpi for lung virus titration (**B**). Immunofluorescent staining of fixed lung tissue from mice that were infected with B/Bris or mock-infected and euthanized at 5 dpi, with cell nuclei (DAPI) labeled in grey, influenza B virus nucleocapsid (IBV/NP) protein in green, Mucin 1 (Muc1) in red, and occludin (Ocln) in blue. Images were captured at 20X (scale bar: 50 μm) (**C**). Average of positive area or mean gray value of IBV/NP protein (**D**), Muc1 (**E**) and Ocln (**F**) in the lungs were quantified. Lungs from mock-infected or IBV-infected males and females were collected at 5 or 7 dpi, sectioned, hematoxylin and eosin (H&E) stained, and scored for perivascular and alveolar inflammation (**G** and **H**). The **#** symbol in the upper right photo highlight proteinaceous fluid in alveolar airspaces - a, alveoli. Arrows in the right upper and lower photo highlight the increased in perivascular cell layers -v, vessels. Data represent the mean ± standard error of the mean (SEM) (n=10–12/group), and asterisks (*) represent significant differences (p<0.05) between the groups based on repeated measures two-way ANOVA or two-way ANOVAs with Bonferroni post-hoc test, or χ2 test for proportion analysis.

To investigate the impact of biological sex on the outcome of IBV infection, adult male and female C57BL/6 mice were intranasally inoculated with B/Brisbane/60/2008 with a PB2 F406Y mutation that enables the virus to productively infect mouse epithelial cells ^23^ (B/Bris; Victoria). After inoculation, mice were monitored for 15 days post infection (dpi) for changes in body mass, rectal temperature, and survival. Significant body mass loss was observed after 5 dpi and returned to the baseline by 14 dpi, with male mice experiencing greater body mass loss and hypothermia than females (Fig. 1a and **Supplementary Fig 1a)**. To determine if male-biased disease severity was caused by a reduced ability to control virus replication, lungs were collected at 3, 5, 7, or 9 dpi for virus titration. Males had higher peak virus titers at 3 and 5 dpi and slower virus clearance from their lungs than females, consistent with increased disease severity in male (Fig. 1b). By 7 dpi, only 1/10 females had detectable infectious virus in their lungs, whereas 6/10 males had detectable virus in their lungs, with all mice having virus cleared by 9 dpi.

We examined the expression of mucin-1 (Muc1) and occludin (Ocln) through immunofluorescence assay (IFA) (Fig. 1c) because Muc1 and Ocln are determinants of mucosal and epithelial barrier function, respectively^24, 25^ that could be sex differentially affected by IBV infection. The average total area for IBV nucleoprotein (NP) expression per lung was significantly greater in males than females at 5 dpi further confirming the greater viral load in males compared to females (Fig. 1d). Within the mock controls, Muc1 expression was higher in female than male lungs. At 5 dpi, IBV significantly reduced the Muc1-positive area in both sexes (Fig. 1e). Expression of Ocln, measured specifically surrounding the airway passage, decreased with IBV infection in both sexes at 5 dpi (Fig. 1f). Pulmonary tissue damage was assessed by evaluating vasculitis and alveolitis through histopathological scoring using hematoxylin and eosin (H&E)-stained lung sections.^17^ Males had greater cumulative histopathological scores than females at 5 and 7 dpi, indicating that greater tissue damage in males corresponded with greater virus loads (Fig. 1g–h). These data suggest that males experience worse outcomes from IBV than females in part because of an inability to control virus replication. Improved mucosal barrier function in the lungs of females prior to infection may contribute to better outcomes.

### Adult males have greater inflammatory and type I IFN responses during IBV

**Figure 2:**
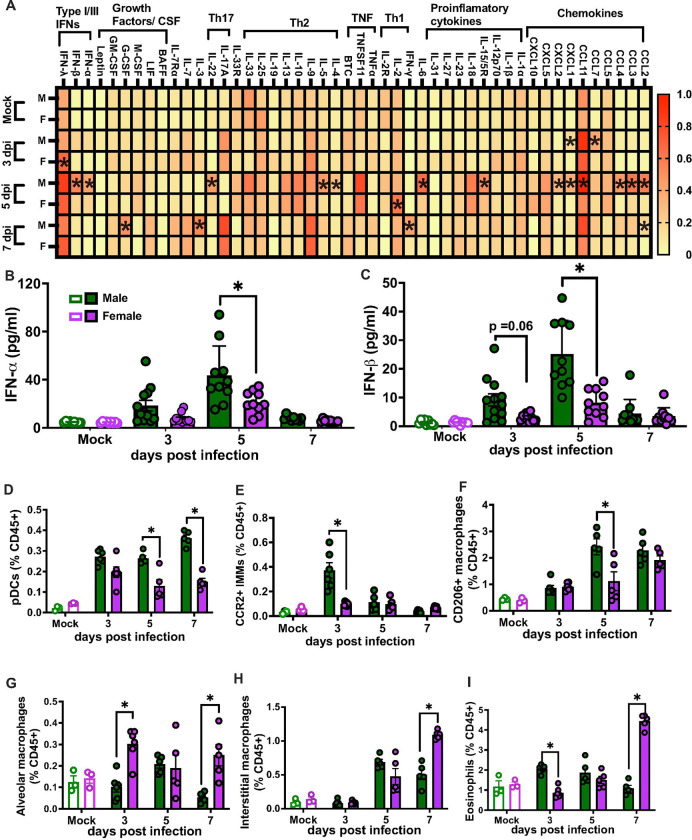
Adult males have greater pulmonary inflammation and type I IFN responses during infection. Cytokines and chemokines were measured in lung homogenates collected from mock- and influenza B virus (IBV)-infected male and female mice at 3, 5, or 7 days post infection (dpi) with B/Bris (**A**). A heatmap was generated by scaling each analyte from 0 to 10 to account for the wide variation in concentrations, facilitating visualization. Asterisks (*) indicate statistical significance, which was determined using the concentrations. IFN-ɑ, IFN-β, and IFN-λ were measured by ELISA in lung homogenates collected from mock- and IBV-infected male (green) and female (purple) mice at 3, 5, or 7 dpi with B/Bris (**B** and **C**). Single cell suspensions from lungs of mock- and IBV-infected males and females were analyzed by flow cytometry for frequencies of PDCA1+ pDCs **(D)**, CCR2+ IMMs (**E**), CD206+ macrophages (**F**), CD11c+F4/80+ alveolar macrophages (**G**), CD11c- F4/80+ interstitial macrophages (**H**), and eosinophils (**I**) at 3, 5, or 7 dpi, expressed as a percentage of live CD45+ cells.. Data represent the mean ± SEM (n=10–12/group), and asterisks (*) represent significant differences (p<0.05) between the groups based on two-way ANOVAs.

In response to IAV, females have greater pulmonary inflammatory cytokine responses than males which contributes to immunopathology and worse outcomes in females compared with male.^17, 26^ Because males suffer worse outcomes than females from IBV, we sought to determine if IBV induced greater pulmonary cytokine and chemokine responses in males. Pulmonary cytokine and chemokine profiles were analyzed in lungs collected from mock-infected mice and from IBV infected mice at 3, 5, or 7 dpi. Of the 48 analytes measured, 42 were detectable in the lungs. Of the 42 analytes that were detectable in the lungs, 19 were differentially expressed between males and females during IBV infection, with 14 being found in greater concentrations in the lungs of males compared with females. At 3 dpi, only CXCL1 and CCL7 concentrations were greater in males than females, but by 5 dpi, considerably more analytes were increased in the lungs of males compared with females, including IL-4, IL-5, IL-6, IL15/15R, IL-22, CCL2, CCL3, CCL4, CCL11, CXCL1, CXCL2, and CXCL2 (Fig. 2a and **Supplementary table 1**). By 7 dpi, only IFN-γ and IL-3 were higher in lungs of males than females (Fig. 2a). In contrast to the robust induction of cytokines and chemokines at the site of infection, there was minimal IBV-induced induction in the spleen and serum, with marginal sex differences noted (**Supplementary Fig. 1b-c**). Type I IFNs (i.e., IFN-α and IFN-β) were also measured in lung homogenates from mock and IBV-infected males and females. While IFN-α and IFN-β were induced by IBV in both males and females compared to mock-infected mice, infected males had greater concentrations of type I IFNs than females particularly at 5 dpi (Fig. 2b–c).

To determine if sex differences in inflammatory cytokine responses were associated with differences in innate cellular responses to IBV, single cell suspensions were generated from mock and IBV infected mouse lungs at 3, 5, or 7 dpi. Male lungs had greater proportions of plasmacytoid dendritic cells (pDCs) at 5 and 7 dpi (Fig. 2d). Frequencies of Ly6C+CCR2+ inflammatory monocytes at 3 dpi (Fig. 2e) and CD206+ macrophages at 5 dpi (Fig. 2f) were greater in male than female lungs. In contrast, females had greater proportions of CD11c+F4/80+ alveolar macrophages at 3 and 7 dpi (Fig. 2g), CD11c- F4/80+ interstitial macrophages at 7dpi (Fig. 2h), and eosinophils at 3 and 7 dpi (Fig. 2i).

To determine if the observations made with B/Bris (Victoria lineage) were unique to a Victoria lineage virus or observed in response to other IBV viruses, adult male and female C57BL/6 mice were infected with B/Wisconsin/1/2010 (B/Wisc; Yamagata-like virus), which carries amino acid mutations in the cap binding domain of the PB2 polymerase subunit, at the same dose as B/Bris. ^23^ Consistent with B/Bris, infection with B/Wisc resulted greater morbidity, pulmonary viral loads, and pulmonary type I IFN responses in adult males than females (**Supplementary Fig. 3a-d).** These data illustrate that while the Victoria virus was more virulent than the Yamagata-like virus used, in each case, males suffered more severe disease and were less able to clear virus from their lungs than females. Subsequent studies were conducted using only B/Bris because only Victoria lineage viruses currently circulate.

### Sex differences in IBV pathogenesis are driven by elevated viral loads and mitigated by early oseltamivir treatment in males

Because IBV induced greater pulmonary antiviral and inflammatory responses in males than females, we next sought to determine if type I IFNs caused tissue damage and disease in males ^27^ or were a consequence of elevated virus replication in males. If elevated type I IFN signaling in males caused more severe IBV outcomes, then pharmacologically inhibiting IFN-αβ receptors (IFNAR) using IFNAR blocking antibodies ^28^ should mitigate disease, particularly in males. Blocking IFNAR in adult male and female mice prior to B/Bris infection resulted in IBV outcomes that were more severe than untreated animals in both males and females. IFNAR blocking resulted in greater morbidity and virus titers resulting in mortality in both males and females **(Supplementary Fig. 4a-d**). Further treatment with IFNAR blocking antibodies starting 2 days post infection (dpi) also did not improve the outcomes in males (**Supplementary Fig. 4e**), highlighting that type I IFNs did not cause sex differences or the more severe outcomes in males.

**Figure 3:**
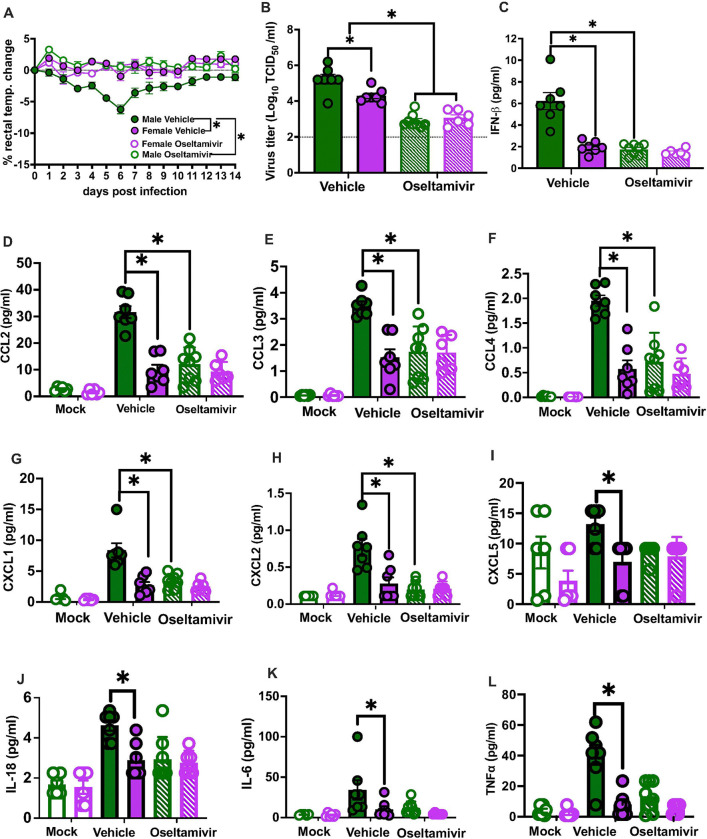
Greater virus replication in the lungs of adult male compared to females causes greater disease pathogenesis in males during influenza B virus (IBV) infection. Adult (8-weeks old) male (green) and female (purple) C57BL/6 mice were treated with vehicle or oseltamivir phosphate through oral gavage starting at 4hr prior and continuing through 5 days post infection (dpi), followed by intranasal inoculation with 10^5^ TCID_50_ B/Brisbane/60/2008F406Y. For morbidity, rectal temperature changes were recorded daily up to 14 dpi (**A**). Subsets of mice were euthanized at 5 dpi for lung virus titration (**B**), type I IFN responses (**C**), and inflammatory cytokines/chemokines responses (**D-L**). Data represent the mean ± SEM (n=10–12/group), and asterisks (*) represent significant differences (p<0.05) between the groups based on repeated measures two-way ANOVA or two-way ANOVAs with Bonferroni post-hoc test.

If elevated type I IFNs were a consequence of greater virus replication in males, then mitigating pulmonary viral load in males might reduce type I IFN activity and improve outcomes, particularly in males. Adult male and female mice were treated with oseltamivir phosphates by oral gavage immediately before IBV infection and continuing through 5 dpi. ^29^ Treatment with oseltamivir prior to IBV infection reduced morbidity (Fig. 3a), lung viral titers (Fig. 3b), and pulmonary concentrations of IFN-β (Fig. 3c) in male, but not female, mice. Treatment with oseltamivir also reduced pulmonary concentrations of cytokines and chemokines, including CCL2, CCL3, CCL4, CXCL1, CXCL2, CXCL5, IL-6, IL-18, and TNF-α (Fig. 3d–l), in IBV-infected male, but not female, mice. Oseltamivir treatment had no discernable effect on IBV outcomes in females, but improved outcomes in males to levels consistent with untreated IBV-infected females. These data illustrate that elevated viral loads in males compared with females cause sex differences in IBV pathogenesis.

### Males experience worse IBV outcomes than females regardless of age

**Figure 4:**
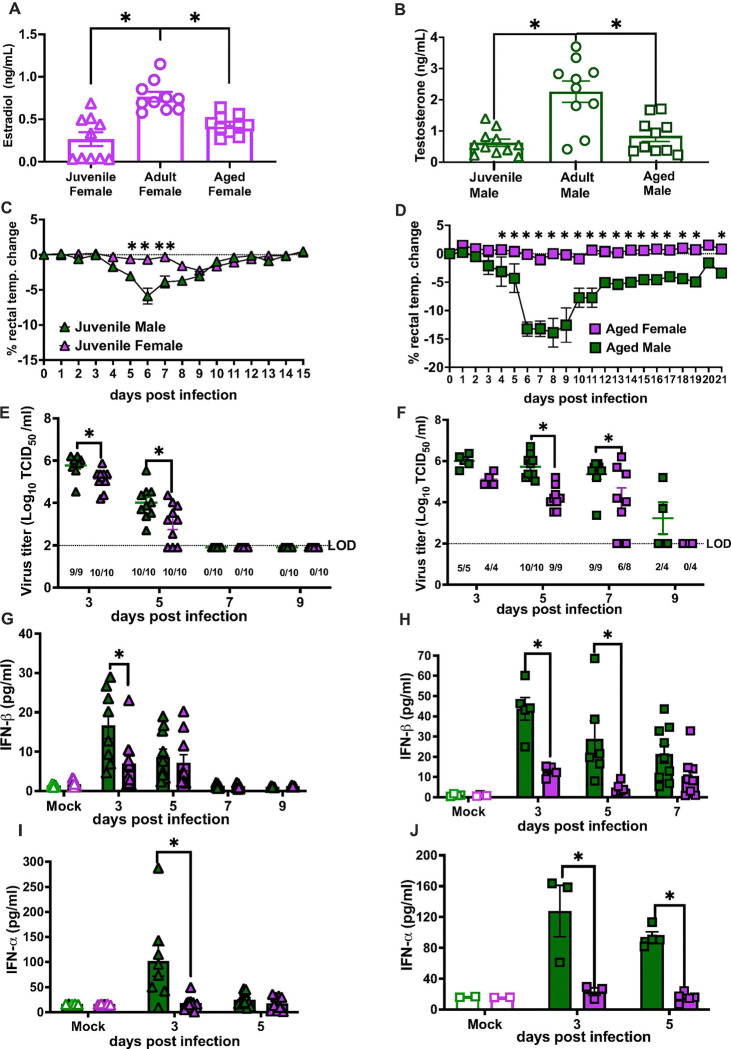
Among both juvenile and aged mice, males suffer worse outcomes than females from influenza B virus (IBV) infection, regardless of gonadal steroid concentrations. Plasma samples were collected from adult (8-weeks old), juvenile (3–4 weeks) and aged (68 weeks) C57BL/6 mice, and concentrations of estradiol in females (**A**) and testosterone (**B**) in males were measured. Juvenile (3–4 weeks) and aged (68 weeks) male and female C57BL/6 mice intranasally inoculated with 10^5^ TCID50 B/Brisbane/60/2008F406Y. For morbidity, rectal temperature changes were recorded daily up to 14 days post infection (dpi) for juvenile mice (**C**) and for 21dpi for aged mice (**D**). Subsets of mice were euthanized at 3, 5, 7, or 9 dpi for lung virus titration for juvenile (**E**) and aged (**F**) mice. Type I IFN responses were measured in lung homogenates from mock and IBV-infected juvenile (**G-I**), and aged (**H-J**) mice. Data represent the mean ± SEM (n=3–12/group), and asterisks (*) represent significant differences (p<0.05) between the groups based on repeated measures two-way ANOVA or two-way ANOVAs with Bonferroni post-hoc test. Triangles represent juvenile mice, circles represent adult mice, and squares represent aged mice.

To determine if sex differences in IBV outcomes were age-dependent, juvenile (3–4 weeks) and aged (68 weeks) males and females were infected with B/Bris and monitored for outcomes for up to 21 dpi. Plasma concentrations of estradiol in females (Fig. 4a) and testosterone in males (Fig. 4b) showed that adult males had higher circulating testosterone than juvenile or aged males, and adult females had higher estradiol than juvenile or aged females. Similar to adult mice, juvenile (Fig. 4c), and aged (Fig. 4d), males experienced greater morbidity than age-matched females, with juveniles experience less disease and recovering faster than aged mice. Among both juvenile and aged mice, males had greater pulmonary virus titers than age-matched females (Fig. 4e–f), with juvenile mice clearing virus faster than aged mice. Among aged mice, females, but not males cleared virus by 9 dpi (Fig. 4g). Finally, among juvenile and aged mice, males had greater pulmonary concentrations of IFN-β (Fig. 4g–h) and IFN-α (Fig. 4i–j). The kinetics of type I IFN responses were age-dependent, with peak responses limited to 3 dpi in juvenile mice but extended 3–5 dpi among aged mice. Because sex differences in IBV pathogenesis were not age-dependent, this suggested that the sex difference was not mediated by gonadal steroids because the patterns of IBV pathogenesis were not consistent with the age-associated differences in concentrations of estradiol in females (Fig. 4a) and testosterone in males (Fig. 4b). If sex differences in IBV pathogenesis were caused by gonadal steroids as they are in IAV^18, 26^, then sex differences in IBV pathogenesis would have been greatest at ages in which concentrations of gonadal steroids were highest (i.e. among reproductive age adults).

**Figure 5:**
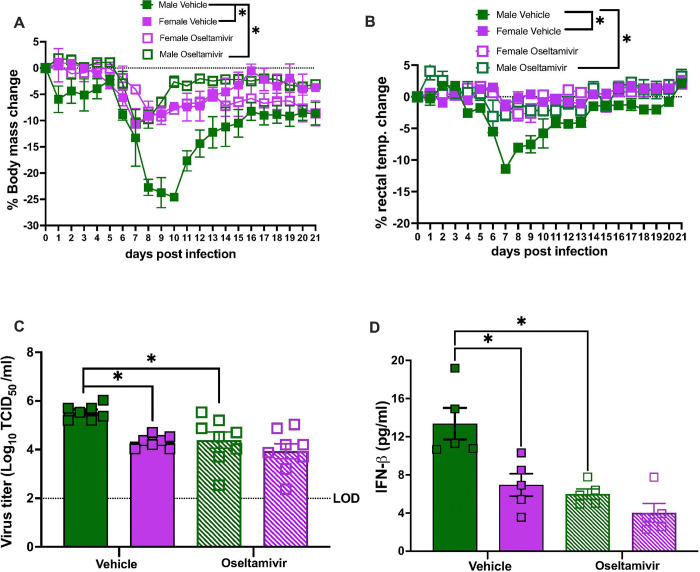
Dose-dependent treatment of aged mice with oseltamivir eliminates sex differences in influenza B virus (IBV) pathogenesis. Aged (68 weeks) male (green) and female (purple) C57BL/6CR mice were treated with vehicle or oseltamivir phosphate via oral gavage 4hr prior of intranasal infection with 10^5^ TCID_50_ B/Brisbane/60/2008F406Y and twice daily for 5 days at a dose of 20 mg/kg/day. Body mass loss (**A**) and rectal temperature changes (**B**) were recorded daily up to 21 dpi for mice treated with oseltamivir at 20 mg/kd/day. Subsets of mice treated with oseltamivir at a dose of 20 mg/kd/day were euthanized at 5 dpi for lung virus titration (**C**) and type I IFN responses (**D**). Data represent the mean ± SEM (n=3–10/group) and asterisks (*) represent significant differences (p<0.05) between the groups based on repeated measures two-way ANOVA or two-way ANOVAs with Bonferroni post-hoc test.

To confirm that at other ages the sex difference was driven by greater viral loads in males than females, aged mice were treated with the same dose of osteltamivir as adult mice ^29^ prior to IBV infection (**Supplementary Fig. 5a**). Aged mice required a higher dose of osteltamivir than adult mice for inhibition of virus replication because administration of 10mg/kg/day failed to reduce pulmonary virus load in either aged males or females (**Supplementary Fig. 5a**). Treatment with 20mg/kg/day of osteltamivir prior to IBV infection of aged mice improved morbidity (Fig. 5a–b), reduced pulmonary viral titers (Fig. 5c), and diminished pulmonary type I IFN responses (Fig. 5d), with the effect size being greater for infected males than females as compared with their vehicle-treated counterparts. These data highlight that the more severe IBV outcomes in males than females are driven by an inability to control virus replication in the respiratory tract and is conserved across the life course.

### Presence of chromosome Y (ChrY) causes male-biased susceptibility to IBV

**Figure 6:**
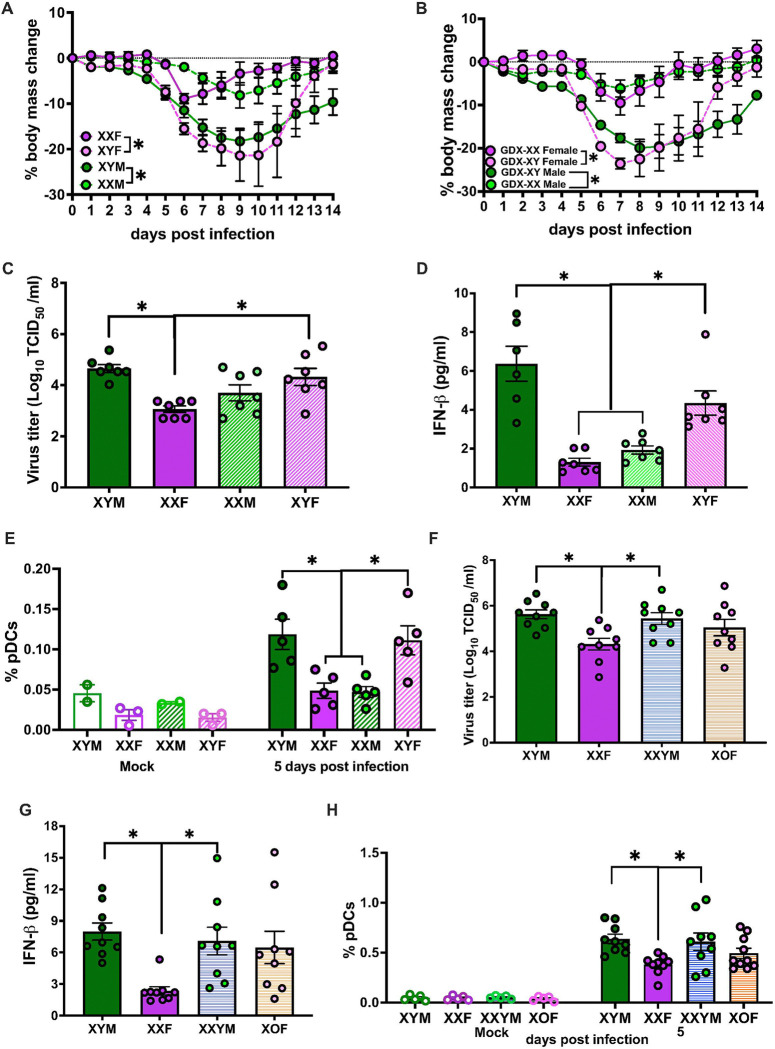
The presence of chromosome Y (ChrY) causes severe influenza B virus (IBV) disease. Eight- to 10-week-old four core genotype (FCG) C57BL/6J mice with ovaries (XXF and XYF) or testes (XYM and XXM) were intranasally inoculated with 10^5^ TCID_50_ B/Brisbane/60/2008F406Y. For morbidity, body mass (**A**) was recorded daily up to 14 days post infection (dpi). In a separate cohort, mice that recovered from gonadectomy (Gdx) were infected with B/Bris, and body mass changes (**B**) were monitored for 14 dpi. Subsets of mock and IBV infected FCG mice were euthanized at 5 dpi for lung virus titration (**C**), pulmonary type I IFN response (**D**), and flow cytometry analysis for PDCA1+ pDCs frequencies in lungs (**D**). XY* mice on a C57BL/6J background, consisting of gonadal females (XX/X0) and gonadal males (XY/XXY), were mock or IBV-infected and euthanized at 5 dpi for lung virus titration (**F**), type I IFN responses (**G**), and PDCA1+ pDC frequencies (**H**). Data represent the mean ± SEM (n=3–10/group), and asterisks (*) represent significant differences (p<0.05) between the groups based on one-way ANOVA or repeated measures two-way ANOVA or two-way ANOVAs with Bonferroni post-hoc test.

Because gonadal steroids did not appear to mediate sex differences in IBV pathogenesis, we sought to uncover the role for sex chromosome complement (i.e., XY vs. XX). Four core genotype (FCG) mice, in which the testes determining factor gene, *Sry*, is transferred from ChrY to Chr3, were created by mating an XX females with an XY^-*Sry*^ males resulting in XX and XY offspring that have either testes (XYM or XXM) or ovaries (XXF or XYF).^30^ FCG mice were intranasally inoculated with B/Bris and monitored for 14 dpi for changes in body mass, rectal temperature, and survival. XY mice, regardless of whether they had testes or ovaries, experienced greater body mass loss (Fig. 6a) and hypothermia **(Supplementary Fig. 6a)** than XX mice. To rule out the activational effects of gonadal steroids as a driver of sex-specific IBV outcomes, FCG mice were gonadectomized. Gonadectomized XY mice experienced greater body mass loss (Fig. 6b) and hypothermia (**Supplementary Fig. 6b**) than XX mice. Among FCG mice, mortality was only observed in XY mice, with XYF mice experiencing greater mortality from IBV infection than XXF mice (**Supplementary Fig. 6c**).

Subsets of FCG mice were euthanized at 5 dpi to measure pulmonary virus titers and immune responses. XY mice, regardless of gonadal sex, had greater lung viral titers than XXF mice (**Supplementary Fig. 6d**). While XXM mice had lower viral titers than XYM, the differences did not reach statistical significance. XY mice with either testes (XYM) or ovaries (XYF) had greater pulmonary type I IFN responses (**Supplementary Fig. 6e**). Because a 3.2 MB region of the X chromosome (ChrX) was translocated to ChrY with *Sry* deletion in our FCG mouse colony, which resulted in the overexpression of some X-linked genes, including *Tlr7* in XY as compared with XX tissues ^31^, we rederived our FCG mice with JAX breeders that did not have this additional translocation. Consistent with the previous FCG mice, XY mice, regardless of gonadal sex, had greater lung viral titers (Fig. 6c) and pulmonary type I IFN responses (Fig. 6d) than XX mice, regardless of gonadal sex. These data suggest that regardless of the expression levels of *Tlr7*, XY mice suffer worse IBV outcomes than XX mice. XY mice (XYM or XYF) also had greater frequencies of pDCs (Fig. 6e) than XXF mice in lungs at 5dpi. Further, XXM mice showed lower frequencies of pDCs in their lungs than XYM mice (Fig. 6e). In contrast, inflammatory CCR2+Ly6C+ monocytes in lungs (**Supplementary Fig. 6f**) were lower in mice with ovaries (XXF and XYF) than mice with testes (XYM and XXM), regardless of their sex chromosome complement, highlighting that these cells likely do not mediate sex differences in IBV pathogenesis.

A limitation of the FCG mice is that the presence of ChrY cannot be separated from the effects of a single dose of ChrX. To investigate the effects of ChrY in combination with either a single or double dosage of ChrX, XY* mice ^32, 33, 34^ were used. In this model, XY* males have a mutated pseudoautosomal region on ChrY (Y*) resulting in abnormal recombination with ChrX and when bred with wildtype females result in gonadal female offspring that are XX (XXF) or X0 (XY^*X^; denoted X0F) and gonadal male offspring that are XY (XYM) or XXY (XX^*Y^; XXYM). XY* mice were infected with B/Bris and at 5 dpi, mice with a double dosage of ChrX and absence of ChrY (XXF) had lower lung viral load (Fig. 6f) and lower level of type I IFN (Fig. 6g) than animals with ChrY (XYM, XXYM), suggesting that the presence of ChrY regardless of the dosage of ChrX are more susceptible to IBV. Infected mice containing ChrY also had greater pulmonary frequencies of inflammatory pDCs (Fig. 6h) and Ly6C+ CCR2+ inflammatory monocytes (**Supplementary Fig. 6g**) than mice with a double dosage of ChrX at 5dpi. Together, these results demonstrate that sex chromosome complement strongly mediates IBV pathogenesis, with the presence of ChrY more than a single dosage of ChrX resulting in greater morbidity, higher pulmonary viral titers, and enhanced type I IFN and pDC responses, regardless of gonadal sex.

### IBV infection suppressed pulmonary *Uty* and *Ddx3y* expression in ChrY-bearing animals

**Figure 7:**
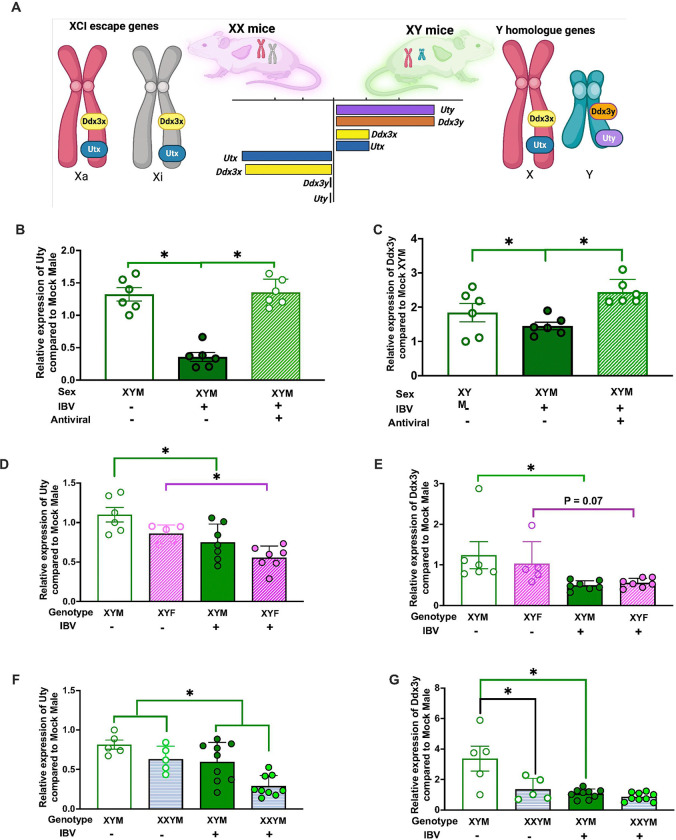
Influenza B virus (IBV) infection alters the pulmonary expression of chromosome Y-linked genes. Schematic illustrating the differential expression of X- and Y-linked genes that may contribute to sex differences in IBV pathogenesis, highlighting conserved X-linked genes in humans and mice (*Ddx3x* and *Kdm6a/Utx*) and their chromosome Y homologs (*Ddx3y*and *Kdm6c/Uty*) (**A**). Illustration depicting differential expression of X- and Y-linked genes in mice were created in BioRender. Chaulagain, S. (2026) https://BioRender.com/8j8bamn(License No. DV29BH7MEN). Wild-type, FCG, and XY* mice were mock-infected or infected with 10^5^ TCID_50_ B/Brisbane/60/2008F406Y and euthanized at 5 dpi for lung collection. RNA isolated from lungs of wild-type XY males was analyzed by qRT-PCR for *Uty* (**B**) and *Ddx3y* (**C**). RNA isolated from lungs of FCG mice with XY chromosomes (XYM or XYF) was analyzed for *Uty* (**D**) and *Ddx3y* (**E**). RNA isolated from lungs of XY* mice carrying a Y chromosome (XYM/XXYM) was analyzed for *Uty* (**F**) and *Ddx3y* (**G**). For all analyses, relative expression was normalized to uninfected XYM controls, with β-actin as the housekeeping gene. Data represent mean ± SEM (n = 5–10 per group). Asterisks (*) indicate significant differences between groups (p < 0.05) by two-way ANOVA.

In XX individuals, ChrX inactivation (XCI) randomly silences one of two ChrX to maintain similar levels of X-linked protein expression between sexes. A subset of X-linked genes (e.g., *Kdm6a*) escape XCI and have higher expression in XX than XY cells.^35^ Greater expression of XCI escapee genes in females compared to males could mediate sex differences in IBV pathogenesis as differential expression of ChrX and ChrY-linked genes can cause sex differences in immune cell function.^36, 37^ Several XCI escapee genes are conserved in humans and mice, including *Ddx3x* and *Utx/ Kdm6a* ^38^, and have the ChrY homologs *Ddx3y* and *Uty/ Kdm6c* (Fig. 7a).Because the presence of ChrY predicted the pathogenesis of IBV, we focused on ChrY-linked genes with ChrX-linked homologs.

RNA was isolated from the lungs of mock and B/Bris infected wild-type males and females at 5 dpi, with a subset of IBV-infected mice treated with oseltamivir. The goal was to determine if greater virus replication drove changes in the expression of these genes. In wild-type males (XYM), IBV infection reduced the expression of *Uty/Kdm6c* (Fig. 7b) and *Ddx3y* (Fig. 7c), which was reversed by oseltamivir treatment. In FCG mice, IBV induced suppression of *Uty/Kdm6c* (Fig. 7d) and *Ddx3y* (Fig. 7e) in the lungs of mice containing ChrY (XYM and XYF), regardless of gonadal sex. In XY* mice, IBV infection reduced the expression of *Uty/Kdm6c* (Fig. 7f) at 5 dpi in the lungs of both XYM and XXYM mice compared to mock controls, regardless of ChrX dosage. In contrast, while infection reduced the expression of *Ddx3y* (Fig. 7g) in XYM, *Ddx3y* expression did not change in XXYM, in part because expression was already low prior to infection in XXYM (Fig. 7g). These results illustrate that IBV infection can reduce ChrY gene expression in the lungs and oseltamivir restores expression to levels observed in mock-infected mice with ChrY.

The X-linked homologs *Utx/Kdm6a* and *Ddx3x* were consistently higher in the lungs of XX mice than XY or X0 mice, regardless of gonadal sex, reflecting their status as XCI escapee genes (**Supplementary Fig. 7a**). In wild-type XX females (XXF), IBV infection reduced *Ddx3x* expression, which was partially restored by oseltamivir, whereas *Utx/Kdm6a* remained below mock-infected levels (**Supplementary Fig. 7a-b**). In FCG mice, expression of both genes in XX mice (XXM and XXF) was largely unchanged following infection (**Supplementary Fig. 7c-d**). In XY* females, following IBV infection *Ddx3x* was reduced in XXF, but not in XXYM, mice. After infection of XY* females, *Utx/Kdm6a* increased in XXF, but remained unchanged in XXYM (**Supplementary Fig. 7e-f**) as compared with mock-infected counterparts. These findings indicate that the expression of the X-linked genes *Utx/Kdm6a and Ddx3x* is greater in mice with a double dose of ChrX, but the effect of IBV infection on the expression of these in lungs was variable.

Together, these findings demonstrate that IBV infection selectively reduces ChrY-linked genes (*Uty/Kdm6c* and *Ddx3y*) in the lung, independent of gonadal sex or ChrX dosage. In mice carrying a ChrY, reduced expression was associated with increased viral replication and more severe disease, highlighting that ChrY-associated transcriptional changes could be a major determinant of sex-biased IBV outcomes.

## Discussion

Influenza B viruses accounting for 20–30% of annual deaths from influenza^21^ with limited epidemiological studies reporting greater rates of hospitalization from IBV infection in males than females.^13, 39, 40^ Using a mouse model of IBV infection, we demonstrate that the males of diverse ages suffer greater morbidity during IBV infection because they are less resistant to infection than females. Across the life course, male mice exhibited greater pulmonary virus replication, delayed clearance, heightened type I and II IFN responses, and increased pulmonary inflammation than females. Sex differences in IBV pathogenesis is primarily mediated by sex chromosome complement rather than gonadal steroids. Consistently across our transgenic and mutant models, mice carrying a ChrY experienced worse disease outcomes, had higher viral loads, and greater pulmonary type I IFNs during IBV infection regardless of ChrX dosage or gonadal tissues. ChrY is a major determinant of IBV pathogenesis.

Greater disease severity in IBV-infected males than females was caused by inability to control viral replication, rather than reduced tolerance to immunopathology. Male mice consistently harbored higher pulmonary viral loads which preceded pulmonary type I IFN induction and inflammatory cytokine responses as well as recruitment of pDCs into the lungs. Pharmacological inhibition of IFN signaling worsened disease in both sexes, indicating that IFN-I responses were protective rather than pathogenic. Antiviral treatment with oseltamivir improved outcomes in males by reducing viral replication which in turn reduced type I IFN response and inflammatory cytokine responses to levels observed in females. How males have greater viral burden requires further investigation and could reflect the reduced mucus production prior to infection.

Using diverse mouse models, sex differences in IAV are largely mediated by gonadal steroid-dependent modulation of immunopathology.^41, 42, 43, 44, 45, 46^ In response to IBV infection, sex differences are largely mediated by sex chromosome complement impacting resistance to infection, with ChrY reducing and a double dosage of ChrX increasing control of IBV replication. IBV replication inhibited pulmonary expression of the Y-linked genes *Uty/Kdm6c* and *Ddx3y* even in the presence of ChrX and ovaries. Virus replication mediated reduced expression of ChrY-linked genes because the effects were reversed by antiviral treatment. Although the X-linked homologs *Utx/ Kdm6a* and *Ddx3x* were expressed at higher baseline levels in XX mice, consistent with their escape from ChrX inactivation, infection-induced changes in their expression were variable and less strongly linked to IBV pathogenesis. Both *Uty* and *Ddx3y* have immunoregulatory functions that may potentially contribute to sex differences in IBV outcomes. *Uty* shares substantial homology with the histone demethylase *Utx/Kdm6a* and has been implicated in modulating proinflammatory responses, with reduced *Uty* expression associated with upregulation in chemokine production and greater pulmonary inflammatory responses, suggesting its protective role in males.^47, 48^ Members of the DDX3 family regulate RNA metabolism, translation, and antiviral signaling, with *Ddx3x* known to participate in both viral replication and type I IFN induction.^49^ Although *Ddx3y* was historically considered testis-restricted, emerging evidence supports broader tissue expression and potential functional redundancy with *Ddx3x.*^49, 50^ IBV-induced suppression of *Ddx3y* may therefore impair antiviral defense in XY cells, potentially contributing to increased viral replication and inflammation. This suppression of *Uty* and *Ddx3y* was reversed by antiviral treatment, indicating that active viral replication is associated with reduction in transcription of ChrY genes. In summary, we have identified a selective vulnerability of ChrY to IBV which appears to underlie male-biased IBV pathogenesis.

Females exhibited greater baseline expression of the mucosal barrier protein, Muc1, which has been shown to limit influenza severity by reinforcing epithelial defenses and dampening TLR-driven inflammation.^24, 25, 51^ IBV infection reduced expression of both Muc1 and the tight junction protein Ocln in males and females, indicating compromised epithelial integrity in both sexes. Higher Muc1 expression prior to infection in females may provide an initial advantage in restricting viral spread; the direct contribution of Muc1 to differential viral replication during IBV infection needs further investigation. Although human studies have not reported sex differences in baseline Muc1 expression in healthy respiratory tissues, analyses of early-stage lung adenocarcinoma samples have shown a trend toward higher Muc1 transcript levels in women than men.^52^ Muc1 expression is also known to be modulated by sex hormones, particularly estrogen and progesterone, raising the possibility that hormonal regulation may impact epithelial barrier function in humans.^53^

This study has several limitations that should be acknowledged. First, although we observe consistent suppression of Y-linked genes during IBV infection, these findings do not establish a direct causal role for these genes in disease pathogenesis. Genetic manipulation of *Uty* and *Ddx3y* in relevant immune and epithelial cell populations will be necessary to define their precise roles in IBV pathogenesis. While antiviral treatment reversed the effects of infection on ChrY gene expression, we have not established which IBV proteins might contribute to loss of ChrY. Our studies were conducted using mouse models and whether similar sex chromosome dependent mechanisms operate in humans remains unknown as we have yet to identify whether people with differences in sexual development, e.g., patients with either Turner (X0) or Klinefelter (XXY) Syndrome are more or less susceptible to IBV than their XX female or XY male comparators. Finally, our study used C57BL/6 mice with a defective Mx1 allele, which limits IFN-mediated antiviral responses and may underestimate IFN potency during IBV infection.^54, 55^ Nevertheless, even in this background, IFN induction was protective, as its inhibition increased disease.

Despite these limitations, our findings provide a conceptual framework for understanding sex differences in susceptibility to IBV. The observation that IBV suppresses the expression of protective genes ChrY, leading to impaired viral control, heightened innate immune activation, and exacerbated pulmonary pathology in XY hosts has not been shown for any virus. These results underscore the importance of considering sex chromosome complement as a biological variable in infectious disease research and suggest that sex-specific host factors may influence responses to antiviral therapies. Elucidating how viruses alter host gene expression as well as how sex chromosome–encoded genes shape antiviral immunity may ultimately inform more precise approaches to preventing and treating influenza in both sexes.

## Materials and Methods

### Viruses and cells

Viral seed stocks of the Victoria (B/Brisbane/60/2008) and Yamagata lineages (B/Wisconsin/1/2010) viruses were generated by reverse genetics by Dr. Daniel Perez (University of Georgia, Athens) as previously described.^56^ Yamagata virus consists of HA and NA from B/Wisconsin/1/2010 and other internal genes from B/Brisbane/60/2008.^23^ Both viruses carry single amino acid mutations in the cap binding domain of the PB2 polymerase subunit (PB2 F406Y) to increase virulence in mice ^23^ Working stocks were generated by infecting Madin-Darby canine kidney (MDCK) cells as described ^57^ and stored in aliquots at −70°C.

### Animals

Adult (8–12 weeks) and juvenile (3 weeks) male and female C57BL/6CR mice were purchased from Charles River Laboratories. Aged (68 weeks) male and female C57BL/6CR mice were obtained of National Institute on Aging. FCG male breeder mice (with or without a 3.2 MB region of the X chromosome was translocated to the Y chromosome) on a C57BL/6J background from Jackson Lab and mouse lines were maintained in house by mating wildtype (XX) females to produce offspring that were: gonadal females (XX and XY chromosomes) and gonadal males (XY and XX chromosomes). XY* breeding pairs on a C57BL/6J background (MMRRC strain 043694-UCD) were obtained from Arthur Arnold (University of California, Los Angeles, USA) and mouse lines were maintained in house by mating XY* male mice that contain an altered pseudoautosomal region (PAR) on Chromosome Y (denoted Y*, composed of complete PAR, no NPY, and a small subsegment of NPX) with wildtype females on a C57BL/6J background to produce offspring that were: gonadal females (XX and X0 chromosomes; XXF, X0F) and gonadal males (XY and XXY chromosomes, XYM, XXYM). Genotypes were confirmed at or after weaning by digital polymerase chain reaction (PCR) analysis for the number of TLR7 and RPP30 copies.

### Biosafety and Ethics

All experiments were performed in compliance with the standards outlined in the National Research Council’s Guide to the Care and Use of Laboratory Animals. All animal procedures were approved by the Johns Hopkins Animal Care and Use Committee (MO21H236 and MOH24158). All efforts were made to minimize animal suffering. All animals were housed at up to 5 mice per microisolator cage under standard biosafety level 2 housing conditions, with food and water provided ad libitum. Work with influenza B viruses in mice was approved by the Johns Hopkins Biosafety Office (P0609250119).

### Animal infection and processing

Mice were anesthetized with ketamine/xylazine cocktail (80 mg/kg ketamine, 5 mg/kg xylazine) injected intraperitonially and inoculated intranasally with 10^5^ TCID50 units of B/Brisbane/60/2008 F406Y (Victoria lineage) or B/Wisconsin/1/2010 F406Y (Yamagata lineage) in 30uL of the virus in Dulbecco’s modified Eagles Medium (DMEM). For morbidity studies, body mass and rectal temperature changes were recorded daily for up to 14 −21 days post infection (dpi) depending on age. Subsets of mice were euthanized at 1, 3, 5, 7, or 9 dpi. Mice were anesthetized using ketamine–xylazine cocktail before either retroorbital or intracardial terminal bleeding. Blood was collected into heparinized tubes, and the serum was separated from blood by centrifugation at 9,600 × *g* for 30 min at 4°C. Serum was stored at −80°C and used to measure cytokines as well as steroids. Lungs and spleen were collected, snap-frozen, and stored at −80°C. When mouse studies were conducted with B/Wisconsin/1/2010 F406Y, Yamagata lineage viruses were still designated BSL2.

### Tissue homogenization and infectious virus quantification

Frozen lungs were homogenized in DMEM. Homogenate was clarified by centrifugation at 12,000 rpm and stored at −80°C until analysis. Infectious virus titers in tissue homogenate were determined by TCID50 assay, as described previously ^58^ through serial dilutions in 96-well plates confluent with MDCK cells incubated at 37°C for 6 days with 6 replicates. Following incubation, cells were fixed with 10% neutral buffered formalin overnight, then stained with naphthol blue for visualization and the Reed-Muench method was used to calculate the TCID50 titer for each sample. ^59^

### Histopathology in lung sections

Lungs of mock-infected and IBV-infected mice were inflated and fixed with zinc-buffered formalin (Z-fix, Anatech, MI, USA) under a constant pressure of 25 cmH2O for at least 24 hours. Tissues were embedded in paraffin, cut into 5-μm sections, and mounted on glass slides. Slides were stained with hematoxylin and eosin (H&E) and used to evaluate lung inflammation. Severity of perivascular inflammation was scored on a scale of 0 – 4 (0, no inflammation; 1, 1 cell layer; 2, 2–3 cell layers; 3, 4 −5 cell layers; 4, > 5 cell layers). Severity of alveolar inflammation was scored on a scale of 0–4 (0, no inflammation; 1, increased inflammatory cells in alveoli, septa clearly distinguished; 2, inflammatory cells fill alveoli, septa clearly distinguished; 3, inflammatory cells fill multiple adjacent alveoli, septa difficult to distinguish; 4, inflammatory cells fill multiple adjacent alveoli with septal necrosis). Extent of inflammation was scored separately for perivascular areas on a scale of 0–4 (0, no inflammation; 1, 2%–25% tissue affected; 2, up to 50% tissue affected; 3, up to 75% tissue affected; 4, > 75% of tissue affected). Individual scores were summed to give a cumulative inflammation score.^60^ The sum of these parameters represents the cumulative inflammation score.

### Immunofluorescence

Lungs were paraffin embedded and 5-μm-thick paraffin sections were mounted on charged slides, deparaffinized, and rehydrated. Antigen retrieval was performed using a steamer and a citrate buffer solution (Abcam) for 30 minutes. Slides were rinsed with PBS and treated with TrueBlack Lipofuscin autofluorescence quencher (Biotium) to remove autofluorescence and then blocked with normal goat serum (NGS; Jackson ImmunoResearch, West Grove, PA, USA; 5%) for 1 hour at room temperature. Sections were incubated with Muc1 monoclonal antibody (MA5–11202, ThermoFiser, MH1 (CT2); dilution 1:150), Occludin monoclonal antibody (33–1500; ThermoFisher, OC-3F10; dilution 1:500) and/or IBV Nucleoprotein antibodies (B/Florida/4/2006) (PA5–81758; ThermoFisher, dilution 1:100) at 4°C overnight. Slides were then washed with PBS and incubated with goat anti-rabbit Alexa fluor 488 (A21206; Invitrogen; dilution 1:1000); goat anti-Armenian hamster Alexa fluor 555 (A78964; Invitrogen; dilution 1:1000); goat anti-mouse Alexa fluor 647 (A21235; Invitrogen; dilution 1:1000) secondary antibodies for 1 h. Nuclei were stained with DAPI (ThermoFisher, Waltham, MA, USA; 1 μg/mL) for 4 min, and images obtained using the LEICA Thunder widefield microscope or Nikon Eclipse Ti2-E microscope (Nikon, Minato City, Tokyo, Japan). Quantitative image quantification was performed using ImageJ. Influenza B virus (iBV) NP and Muc1 signals were quantified from whole lung by measuring total positive area. Ocln expression was quantified as mean fluorescence intensity (integrated density normalized to ROI area) from regions of interest surrounding bronchiolar airways. All images were acquired and analyzed using identical imaging and thresholding parameters. Five to eight fields were analyzed per section, and values were averaged per animal prior to statistical analysis.

### Real-Time RT-PCR

Total RNA was harvested from lungs with the RNA-extraction kit (Qiagen). RNA was quantified using a NanoDrop and up to 1 μg was used to synthesize cDNA using High-Capacity cDNA Reverse Transcription Kit (Applied biosystem, 4368814). Taqman probes (*Kdm6c* (*Uty*): Mm00447710_m1, *Ddx3y*: Mm00465349_m1, *Kdm6a*:Mm00801998_m1, *Ddx3x*: *Mm04207948_gH*) were obtained from Thermo Fisher Taqman probe catalog (4331182). Expression was quantified using the QuantStudio-6 Real time PCR system. β-actin was used to normalize the expression of each gene as a housekeeping gene. Fold changes were calculated by comparison to mock XY male for each gene.

### Multiplex Cytokine analysis

Cytokines and chemokines in lungs were measured using the Immune Monitoring 48-Plex Mouse ProcartaPlexTM Panel (Thermo Fisher Scientific EPX480–20834-901) miniaturized with the Curiox DA-Bead DropArray platform (Curiox Biosystem). Following analytes were quantified: granulocyte colony-stimulating factor (G-CSF), granulocyte- macrophage colony-stimulating factor (GM-CSF), macrophage colony-stimulating factor (M- CSF), IFN-a, IFN-g, IL-1a, IL-1b, IL-2, IL-2R, IL-3, IL-4, IL-5, IL-6, IL-7, IL-7Ra, IL-9, IL- 10, IL-12p70, IL-13, IL-15, IL-17A, IL-18, IL-19, IL-22, IL-23, IL-25, IL-27, IL-28, IL-31, IL- 33, IL-33R, Leukemia inhibitory factor (LIF), CXCL1, CXCL2, CXCL5, CXCL10, CCL2, CCL3, CCL4, CCL5, CCL7, CCL11, TNFa, receptor activator of nuclear factor kappa-Β ligand (RANKL), B-cell activating factor (BAFF), Betacellulin, vascular endothelial growth factor (VEGF)-A, and Leptin. Concentrations of samples were calculated by plotting the expected concentration of the standards against net MFI using a 4 PL curve fit. Undetectable values were set to 50% of the lower limit of detection according to the manufacturer.

### IFN-a, IFN-β, and IFN-λ ELISA

IFN-a, IFN-β, and IFN-λ in lung homogenate were measured by ELISA according to the manufacturer’s protocol (PBL Assay Science).

### Steroids measurements

Concentrations of testosterone and estradiol were measured in hormone-extracted serum samples using diethyl ether at a 1:5 sample-to-ether ratio. Testosterone concentrations were measured using mouse IBL Testosterone Kit and Estradiol was measured using the Milliplex multi-species hormone magnetic bead kit (MSHMAG-21K) and quantified following the manufacturer’s instructions.

### Flowcytometry

Lungs were collected from a subset of mock infected mice or mice at 3, 5, and 7 dpi for flow cytometry. Briefly, single cells were prepared from lungs. Enzymatic digestion of lung was performed with lung digestion media containing lung 1^0^ media (RPMI media+ 2.5% FBS + 10mM HEPES) with collagenase II (Gibco 17101015) and DNAse I (Millipore Sigma 4536282001) added. Cells were passed through a 70 μm cell strainer, washed, and subjected to red blood cell lysis (ACK lysis buffer), and Fc blocking anti-CD16/32 (BD Biosciences 554142). Cells were counted using an automated cell counter (Nexcelom). Viability was determined with AO/PI and 2 × 10^6^ live cells used per sample. Single-cell suspensions obtained were stained with surface marker antibody cocktails **(Supplementary Table 4 and 5**) for 30 min in the dark, washed, fixed in 2% paraformaldehyde containing FACS buffer, and acquired on BD Symphony A3 Cytometer (BD Biosciences) and analyzed with FlowJo (version 10.8.1). Gating was performed as described in **Supplementary Fig. 2.**

### Antiviral treatment

Experimental animals were administered vehicle alone (purified water) or Oseltamivir phosphate (Sigma Aldrich). Adult mice were administered treatment via oral gavage 4 hr prior of intranasal infection and twice daily for 5 days at a dose of 10 mg/kg/day.^29^ Aged mice were administered at a dose of 10 or 20 mg/kg/day following similar timeline.

### IFN-ab receptor (IFNAR)-blocking

Experimental animals were treated intraperitoneally with an IFN-α/β receptor–blocking antibody (anti-IFNAR; MAR1–5A3, Bio X Cell) or isotype control (mouse IgG1, clone MOPC21). An initial dose of 1 mg was administered either 1 day prior to infection or 2 days post-infection with B, followed by successive doses of 250 μg on alternate days.^28^ Antibody-treated, B/Bris-infected mice were monitored for changes in body mass and rectal temperature for up to 15 days or until endpoint criteria were met. Subsets of mice were euthanized at 5 dpi for collection of tissues collection.

### Statistical analyses

Statistical analyses were performed using GraphPad Prism v10.0.3 (GraphPad Software) with statistical tests indicated in figure legends. Repeated measures two-way ANOVAs were used for body mass and hypothermia analyses across time, followed by Tukey’s multiple-comparison post-hoc tests. Infectious virus titers, flow cytometry, histopathological assessments, immunofluorescence quantifications, IFN-a and IFN-β levels, steroids, and qt-PCR was analyzed by 1-way or 2-way ANOVAs followed by post hoc Bonferroni post-hoc test. Proportion analysis for viral titer data was performed using χ2 test. Multiplex cytokine quantifications were analyzed using two-way ANOVA followed by Bonferroni *post-hoc* test with correction for multiple comparisons; males and females were compared at each time point, including mock controls. For all data, individual data points are shown for individual experimental mice. Mean or median differences were considered statistically significant at p < 0.05.

## Supplementary Material

Supplementary Files

This is a list of supplementary files associated with this preprint. Click to download.

• JHU79SupplementaryTable1Lungs.docx

• SupplementaryFigures.pdf

• JHU79SupplementaryTable2Spleen.docx

• JHU79SupplementaryTable3Serum.docx

• JHU79SupplementaryTable5.docx

• JHU79Supplementarytable4.docx

## Data Availability

All data will be made publicly available upon publication and upon request for peer review.
